# Experiences and impact of the UK lockdown amongst adults who have a facial visible difference

**DOI:** 10.1007/s12144-022-04089-5

**Published:** 2023-01-26

**Authors:** Philippa Tollow, Claire Hamlet, Fabio Zucchelli, Heidi Williamson, Diana Harcourt

**Affiliations:** Centre for Appearance Research, University of the West of England, Bristol, UK

**Keywords:** Body image, COVID-19, Visible difference, Thematic analysis

## Abstract

Globally, COVID-19 has been shown to have had a wide ranging and significant impact on individuals’ daily living, and physical and mental health. However, there are some groups of individuals who may encounter unique challenges with regards to COVID-19 and whose experiences have not been investigated thus far. Therefore, this study aimed to understand the experiences of adults with a facial visible difference in relation to COVID-19 and lockdown. Semi-structured interviews were conducted with 21 adults with a variety of facial visible differences (e.g., cleft lip/palate, facial scars, skin conditions) and analyzed using inductive reflexive thematic analysis. This analysis generated three themes (Escaping the external gaze; Existing feelings manifesting in new challenges; COVID-19 taking priority). The results of this study suggest that the first U.K. lockdown from the COVID-19 pandemic created significant challenges for some individuals with a facial visible difference, and ongoing restrictions and social distancing measures might be particularly challenging for those who experience anxiety around social encounters. These findings highlight the importance of providing appropriate and accessible support for people with facial differences during lockdown and as restrictions ease.

## Introduction

COVID-19 has had a wide ranging and significant impact on people’s daily lives, and physical and mental health (McBride et al., 2021). Although the full extent of the impact of the pandemic is still unknown, research to date has examined a host of psychosocial issues including pandemic-related anxiety (McElroy et al., 2020), and loneliness (Bu et al., 2020; Groarke et al., 2020). Some groups of individuals may face particular or unique challenges and consequences, and knowing more about the experiences of these groups is important if appropriate support is to be made available. Multiple researchers have considered the impact of lockdown on body image and disordered eating (for example Hunter & Gibson, 2021; Robertson et al., 2021; Rodgers et al., 2020). However, few studies have explored the impact of COVID-19 and lockdown on people with an altered or unusual facial appearance (e.g., cleft lip and palate, facial scars, skin conditions), with those that have explored this topic primarily focusing on specific conditions and healthcare experiences (e.g., Bruce et al., 2021; Costa et al., 2021).

The U.K. Government’s response to the pandemic involved lockdown and social distancing measures that came into force in March, 2020. These included staying at home except for essential activities including seeking medical care and to carry out keyworker duties, e.g., healthcare providers continued to work. In addition, the population as a whole was required to reduce in-person social interaction and maintain two metres’ distance from anyone other than their household members. Public gatherings were cancelled, and businesses and venues such as pubs, restaurants, cinemas and theatres were closed. This resulted in isolation for many. Those who tested positive for COVID-19 or had symptoms of the disease were required to self-isolate, and those deemed clinically extremely vulnerable (e.g., those with health conditions that impact on the immune system), were advised to stay indoors and minimise their interaction with others (‘shielding’). From 24th July 2020, face coverings became mandatory for most people when using public transport and in indoor public places including shops and healthcare settings. Exemptions were allowed for a range of reasons including being unable to “put on, wear or remove a face covering because of a physical or mental illness or impairment, or disability” (Department of Health and Social Care, 2020).

Whilst lockdown, social distancing and social isolation could be testing for anyone (Williams et al., 2020), there are several reasons why people who have a visible facial difference may have unique experiences in these circumstances. First, people who look different to ‘the norm’ often report that the reactions of other people (including staring, unsolicited questioning, teasing, and avoidance) are difficult to handle (Clarke et al., 2014). Lockdown may offer a reprieve from the stress of unwanted public attention, by endorsing social avoidance. Newell’s (1999) fear-avoidance model argued that anticipation of social interactions as a negative experience leads to avoidance of social situations. This is usually deemed a negative outcome, but familiarity with reduced social interaction could prove beneficial for some during lockdown. However, lockdown and social distancing also increase loneliness (Bu et al., 2020; Groarke et al., 2020), reduce in-person opportunities to challenge negative societal reactions to visible difference, and to develop or practice strategies to manage these situations, for example through the use of social skills that are often included in interventions to support people with appearance-related anxiety (Norman & Moss, 2015).

Second, limited opportunities for face-to-face contact and in-person interaction during the pandemic have led to a dramatic increase in virtual interactions (e.g., meetings, webinars, chat, and social events) through online platforms (e.g., Zoom™, Skype™), drawing attention specifically to the face which is now ‘on show’ in a way unlike it had been before. Bailenson (2021) suggested that teleconferencing platforms lead to negative affect and self-evaluation, partly due to the constant “mirror” when users see their reflection unless they choose to ‘hide self view’. This could be a particular challenge for people who are dissatisfied with their facial appearance, regardless of whether or not they have a visible difference, and lead those who are self-conscious or troubled by a facial difference to restrict any videoconference interactions to people they already feel comfortable with (for example family, friends and work colleagues). It should be recognised that many with a visible difference adjust well to their appearance (Egan et al., 2011), and experiences of videoconferencing are likely to vary considerably. However, until now, there has been a lack of research into this aspect of living with a visible difference.

Third, although face coverings became compulsory in many settings, we know nothing about how they might impact on the experiences of people with visible differences affecting the mouth, chin, nose and cheeks (e.g., cleft lip and jaw malocclusion), which are typically hidden by masks. This might offer a temporary reprieve from public gaze, as described above, and widespread use of face coverings by the general population might make some aspects of life ‘easier’ for those with facial differences who might then ‘blend in’ rather than ‘stand out’. Also, Bogart (2021) posited that masks restrict the use of facial expressions in social situations, so people with conditions such as facial paralysis are potentially advantaged by being experienced in alternative expression, for example posture, tone of voice, hand gestures. However, although camouflage or disguise of a visible difference can be an effective strategy in some instances, reliance can be problematic (Clarke et al., 2014) and ‘revealing’ a difference that was previously hidden can be stressful (Sharratt et al., 2020). Removing a facial covering might therefore be an additional source of anxiety. People whose visible difference might make it difficult or impossible to wear a mask (for example, those with the condition Microtia which affects the appearance of ears), may face additional challenges.

Fourth, some facial differences are associated with health conditions or treatments that weaken the immune system (for example, chemotherapy-induced alopecia), rendering those affected clinically extremely vulnerable and requiring them to ‘shield’ and isolate. Heightened levels of psychological distress and loneliness have been reported amongst people who have been shielding (Robinson & Daly, 2020), but no research has specifically examined the experiences of people with a visible difference associated with a health condition. In addition, many planned treatments, such as surgical procedures for those with cleft lip/palate, have been heavily disrupted by the impact of the pandemic (Bruce et al., 2021) and the delivery of care has been altered (for example, burn care follow up via telemedicine rather than face-to-face appointments (Varma et al., 2021)). Furthermore, lack of access to and availability of face-to-face psychosocial support (e.g., one-to-one specialist interventions, and support groups) due to reduced services from charities and healthcare providers, could increase demand and use of online interventions and support (e.g., Bessell et al., 2012; van Dalen et al., 2022).

In summary, the psychosocial consequences of the coronavirus pandemic could present unique issues for people with visible differences, particularly those affecting the face. These are in addition to the anxieties and mental health issues affecting the general population during lockdown. Therefore, this study aimed to understand the experiences of adults with a facial visible difference in relation to COVID-19 and lockdown.

## Methods

### Participants

Twenty-four people enquired about the study in response to the email they had received. Of these, three did not respond to the team’s efforts to arrange an interview. All 21 adults who took part were living in the United Kingdom and identified as having a visible difference that affected their head or face. This included 17 women and 4 men, and ages ranged from 21 to 63 years. They reported their visible differences as being: Cleft Lip and/or Palate (5), Alopecia (5), Microtia (2), Facial Palsy (2), Vitiligo (1), Port Wine Stain (1), Jaw Malocclusion (1), Craniosynostosis (1), Trichotillomania (1), Scarring (1), Congenital Melanocytic Nevi (1), Eczema (1) and Apert Syndrome (1) (note: some participants reported multiple visible differences) (see Table 1 for further demographic details).

**Table 1:** Participant demographic information

		Frequency (n = 21)	Percentage (%)
Gender	Female	17	81%
	Male	4	19%
Ethnicity	White British	18	85%
	White European	1	5%
	British Asian	1	5%
	Indian	1	5%
Employment status	Working	12	57%
	Carer	3	14%
	Student	2	10%
	Unemployed	2	10%
	Retired	2	10%

### Design

Due to the exploratory nature of this study, a qualitative approach was taken, using semi-structured interviews to examine participants’ experiences. Ethics approval was granted by the authors’ host institution (the University of the West of England, Bristol) and the study was performed in accordance with the ethical standards as laid down in the 1964 Declaration of Helsinki and its later amendments or comparable ethical standards. Each participant provided written consent before their interview began.

### Materials

Interview questions were developed by the authors based on their knowledge of the visible difference literature and their previous experience of research in this field. A PI (Participant Involvement) advisor who has personal experience of visible facial difference gave valuable feedback on the draft questions. This feedback was shared with all the authors and a final set of questions was agreed upon. These included questions regarding: the participant’s background and current family/employment circumstances, experiences of social distancing/lockdown, feelings around anticipated lockdown easing, support needs, and any additional issues that the participant wished to discuss. 

### Procedures

An email introducing the study was sent to adults over the age of 18 years who had previously registered with a participant pool at the authors’ institution and had indicated that they had a visible difference. Those who were interested in taking part were invited to contact one of the authors for further details, to ask any questions about the study and arrange an interview if they chose to take part. Participants were given the choice of being interviewed by a male or female researcher, and for their interview to take place over the telephone or video-conferencing (Microsoft Teams™). In-person interviews were not possible due to the COVID-19 restrictions that were in place at the time (May-June, 2020). Participants were offered a £25 online shopping voucher to thank them for sparing their time to take part, in line with recommendations (e.g., Gelinas et al., 2018) that offering financial compensation is reasonable and ethical and potentially increases accessibility of the research to include people who would otherwise not be able to engage with it. All interviews were conducted in English, audio recorded, and transcribed verbatim by a professional transcriber, approved by the author’s University.

Interviews were conducted in English by the authors, depending on availability at the participant’s preferred time and participant preference for a male or female interviewer. None of the researchers have personal experience of a facial difference, but they do all have extensive experience of conducting qualitative visible difference research They were all working from home in response to COVID-19 restrictions when the current study took place. Whilst planning this study, they discussed and reflected on their own thoughts and expectations as to what might be found, and throughout the study they were aware of their influence on the interviews and analysis. They provided debriefing sessions for one another as needed and kept reflective notes throughout the study.

### Qualitative analyses

An inductive reflexive thematic analysis was conducted by the first author, in collaboration with the second author and in discussion with all authors, in order to explore participants’ shared experiences. The ontological and epistemological assumptions of the researchers were carefully reflected upon and, as a result, analysis adopted a critical realist perspective. This approach acknowledges the subjective nature of the knowledge accessible by the researcher throughout the research process and the subjective lens through which they view the data, whilst also assuming the existence of some ‘external reality’ in order to provide a foundation for knowledge and facilitate practical application of the research (Guba & Lincoln, 1994). Acknowledgement of this theoretical underpinning to the research is important in order to understand the lens with which the researchers have analyzed the data and the assumptions present in their analysis (Fletcher, 2016).

Data analysis followed the six-step process outlined by Braun and Clarke (2021): (1) ‘familiarisation’, (2) ‘data coding’, (3) ‘generating initial themes’, (4) ‘reviewing and developing themes’, (5)| ‘refining, defining and naming themes’, (6) ‘writing up’. Details of this process are provided below for transparency. The first and second authors (PT and CH) worked separately on steps 1–3, focusing their analysis on 12 transcripts each, but familiarising themselves with the entire dataset. After working through steps 1–3, they then came together to compare their analysis, working collaboratively on steps 4–5, and then presented initial proposed themes with exemplar quotes to the other authors (FZ, HW and DH). With suggestions from all the authors, the first and second authors then worked together to repeat step 5 until a set of final themes were agreed. Finally, PT and CH worked together to ‘write up’ these themes, with supporting evidence. At each stage, the researchers moved between their interpretations and the data to ensure that the analysis was grounded in participants’ accounts, with records of each stage kept and reviewed by the research team. Reflecting the topic of study, all meetings and collaborative working took place via video-conferencing and collaborative work on shared documents.

## Results

The analysis generated three themes: ‘Escaping the external gaze (subthemes: ‘relief from social pressure’, ‘anxiety around returning to normal’), ‘Existing feelings manifesting in new challenges’, and ‘COVID-19 taking priority’. All themes are presented in detail below with illustrative participant quotes, with pseudonyms used throughout.

### Escaping the external gaze

This first theme captures an important element of lockdown for many participants, who described reduced interactions with the outside world as representing a break from others and reactions to their appearance. Although participants described this as allowing them to escape the external gaze of others, some were also concerned about the negative impact that this may have upon their confidence when the time came to return to normal life. This theme is explored through two subthemes, ‘Relief from social pressure’ and ‘Anxiety around returning to normal’.

### Relief from social pressure

Although some participants had keyworker roles and had continued working as usual, all had experienced a level of social isolation because of lockdown and the need to maintain social distance from others. Whilst many missed in-person interactions with friends and family, they reported that lockdown had also provided an escape from day-to-day interactions that had previously caused them anxiety, particularly due to the reduced number of interactions with people who they were not already familiar with.

*“I don’t go out as much, so I don’t see new people and it’s really around new people I feel the most self-conscious. […] So I think not having to meet a lot of new people has been quite nice” (Rachel)*

Similarly, lockdown also prompted some participants to experiment with their appearance, without the usual concern of drawing attention to their visible difference. In the quote below, Amanda describes how the closure of hairdressers and encouragement from her friends had led her to change her hairstyle, which had previously covered her facial difference.

*I guess it’s making me reconsider, um, you know, I’ve managed to go for, like, what three months or something without having a full fringe … nothing awful has happened to me, uh … [laughs] it should be … it should be okay so … maybe I’ll keep growing it out, maybe I won’t, I’m not sure, but I guess at least something that, I guess, has prompted me to challenge about the way I look. (Amanda)*

However, many also experienced complex feelings regarding this period of relief from social interactions, with some describing associated guilt because of the overwhelmingly difficult issues being faced by so many as a consequence of the pandemic:

*“And I feel quite guilty because I’ve actually seen it as a breath … breath of fresh air for me, just a little mental break” (Jill)*

### Anxiety around returning to normal

Although participants described relief of having fewer interactions with others, there was also a concern that lockdown may have impacted on their social confidence in the longer term, and many reflected on their own concerns about reengaging with the wider world.

*“Because we’ve now been locked up for so long, I do feel a bit like I’m almost reintroducing my burns to the world” (Sarah)*

Some participants acknowledged the role that regular social interactions had played in their previous adjustment to their visible difference and described returning to ‘normal’ with significant trepidation.

*“Back to the exhaustion, back to being seen, back to being on public view 24 hours a day... it’s decreased my anxiety. It’s probably not helping me in the long run because I’m not keeping that interaction, which is keeping my emotions in check.” (Jill)*

In addition, social distancing had presented some participants with a new concern, namely the possibility that using visual cues to give distance to other people in public settings was increasing attention and focus on their face (and therefore their facial difference).

*“I feel like I am keeping out of the way, but obviously then if they’re looking at me I think, well, why are they looking at me because I feel like I’m far enough away from you, or are you looking at me because of my appearance? So, it makes my brain over think more” (Jane)*

This consequence of reduced social interaction during lockdown can be further seen in the contrasting experiences of those who had continued to work during lockdown, due to holding a role as a key worker. For these participants, their relatively regular routines could be seen to have had a protective role and the return to normal was not perceived to be as much of a big issue.

*“I think I would have … probably would have been more aware of it if … if I had to kind of isolate more and then I was starting to go out and see people more, probably, yeah, there would be in my mind focus … um, more then” (Amy)*

In summary this theme ‘Escaping the external gaze’ and its subthemes demonstrate a mixed blessing perceived by many participants as a result of lockdown restrictions. Reduced social interactions was a relief for many and a welcome break from previous anxieties, however, many anticipated anxiety increasing again when returning to normal routines.

### Existing feelings manifesting in new challenges

Participants’ experiences of COVID-19 and lockdown appeared to be strongly influenced by their feelings regarding their visible difference prior to lockdown. For participants, this added a complicating factor to the many changes in behaviour experienced by the general population (e.g., increases in video calling, wearing face coverings, social distancing).

Those who appeared to have a higher level of acceptance and adjustment to their facial difference in their daily life prior to lockdown seemed to manage the new challenges they encountered during this time and appeared to experience few issues in relation to their visible difference.

*“It’s not really affected me at all really […] I don’t really see myself as looking different really, this is just me” (Mark)*

For example, many of the participants who appeared to demonstrate a high level of adjustment to their appearance discussed their increased use of video calls during lockdown in terms of the social benefits this offered, rather than any concerns relating to their visible difference.

*“such an amazing thing to have in a time like this because you can see everybody on that screen and it’s nicer, I think, sometimes to see a face rather than just listen on the phone” (Jackie)*

Similarly, when discussing the potential use of face coverings, these participants tended to discuss masks in terms of safety or practical concerns (e.g., feeling hot or their glasses steaming up); and whilst these participants still experienced challenges related to lockdown, such as missing family members or worry about the risk of contracting COVID-19, their concerns were largely similar to those felt by the general population and seemed, on the whole, unrelated to their visible difference.

In contrast, those who reported previously experiencing significant psychosocial issues in relation to their visible difference seemed to struggle with new challenges associated with lockdown and the COVID-19 pandemic. This group discussed having ongoing concerns related to their facial difference and described how these had manifested in new issues during lockdown. For example, lockdown had led them to become more self-conscious or given them more time to ruminate about their appearance. This was typified through participants’ thoughts and feelings around video calls, with concerns that this form of communication draws attention to facial differences and leads to them feeling uncomfortable.

*“I tend to do phone calls because […] obviously on camera my face shifts and then it goes from looking how I think of as normal and then on my camera it looks completely different. It looks completely unsymmetrical, so I’ve tended to prefer phone calls because obviously that’s just my voice” (Rachel)*

Participants also described how their own attention was drawn more to their appearance through this mode of communication, with their own video featuring on the screen alongside others. Similarly, spending an increased amount of time at home meant that some participants faced more exposure to their own appearance than normal, and described subsequent negative effects on their self-esteem. For example, Sarah spoke of seeing her ‘natural’ appearance more often in a household mirror:

*“It’s definitely had a really negative effect on how I view myself…. [prior to lockdown] I was avoiding mirrors and stuff and I wasn’t … I’d go like weeks without looking in the mirror. I suppose like everybody else I haven’t really been bothering very much with my appearance while I’ve been in lockdown, which I think has maybe been a bit of like my worst enemy” (Sarah)*

This discomfort with an increased focus on their visible difference can also be seen in participants’ discussion of masks. Although all the participants stated that they would wear face coverings in public if required to do so, some were concerned that this would draw attention to them and leave them prone to negative judgement by others.

*“I can’t stand anything that draws attention to you because I don’t need attention drawing to me. I always feel I’ve got a big, big neon sign above my head, um, saying poke me, that’s how society makes me feel” (Jennifer)*

For some participants, feelings of self-consciousness around their appearance had led them to embrace the situation and new ways of exerting an element of control over other people’s perceptions of them. For example, some described how video calls allowed them to control carefully how they presented their appearance to others, and some felt that masks offered some comfort in situations where they would otherwise worry about being judged based on their visible difference.

*“I’m constantly aware of it when I meet people, because I know it’s there, um, and I always think are they picking up on it or what’s their interpretation of me […] that is something that I think about when I first meet someone for the first time. Uh, and as I say, obviously, wearing a face mask, it kind of took … created a barrier and kind of took that away in some respects” (Amy)*

Finally, some participants described lockdown in terms of a sense of physical restriction which brought back difficult memories of situations when they had struggled with feelings of isolation and difficult emotions in the past.

*“when I first had my burn and stuff I didn’t leave the house for a few weeks…So I had to be in hospital and then when I came home I had to be in house isolation for a month and it’s kind of like I’ve repeated that by doing this isolation. So, it’s kind of brought back some of, like, the sort of lonely aspect” (Sarah)*

In summary, lockdown did not appear to alleviate or aggravate most participants’ feelings towards their appearance, but some did describe experiencing a range of reactions to lockdown and these could be seen to reflect their existing feelings around their visible difference.

### COVID-19 taking priority

Many participants described the global pandemic as having dominated people’s lives around the world, with some describing how this had reduced focus on their own facial difference during this time and therefore also reduced their concern around their visible difference. For example, suggesting the changes to their lives as part of the fight against COVID-19 had consumed much of their attention in recent months.

*“you could say, in these times it’s [visible difference] the least of my worries if you like [laughs]” (Jackie)*

However, participants also expressed concerns about the ongoing impact that the emphasis on ‘fighting’ COVID-19 would have on resources for treatment and support for those living with a visible difference. Whilst most participants did not have conditions that made them more susceptible to COVID-19, some did express concern about the changes that were already taking place with regards to care that they or others were receiving, or the possibility that this would be affected in the future.

*“Are we going to not have money to pay for people like me having their treatment because we’ve spent so much money on this disease? Is the waiting list going to be so long that I might have to wait way longer than I think I will have to wait? And, as a result of that, is there … and there is … a likelihood that the, um, part of my, um, dental work that hasn’t been touched but can break, what happens then? Um, so it’s … it’s things to do with me personally and how future treatment might be impacted by funding and time and stuff like that” (Julie)*

Similarly, some were worried about the impact that COVID-19 could have on the provision of support from the charitable sector to those living with a facial difference:

*“I think we’re doing a lot less awareness around this kind of thing [visible difference] because obviously it’s now a non-essential service so we’ll get pushed to the back of the queue really, which in times of pandemic makes sense but, um, people need to access the services that might be difficult. I think it’s … it’s understandable some things are essential and some things are not. But then, just because something’s not essential it doesn’t mean it’s not important.” (Rachel)*

This difference between ‘essential’ and ‘important’ services reflects the view of many participants, who acknowledged the key role that medical treatment and/or psychological support had played in their wellbeing and were concerned about the impact on others if they were not able to access the same resources in a timely manner.

In summary, this theme can be seen to demonstrate another complexity of individuals’ experiences during this time, with participants reporting both positive and negative repercussions of their focus being directed away from visible difference. Some participants identified a personal benefit to their attention having been on the pandemic, others were concerned about the impact that this could have at a larger societal level.

## Discussion

This study aimed to explore the experiences of people with a facial difference during the COVID-19 pandemic and U.K. lockdown. The analysis resulted in three themes: ‘Escaping the external gaze’ (subthemes: ‘Relief from social pressure’, ‘Anxiety around returning to normal’), ‘Existing feelings manifesting in new challenges’, and ‘COVID-19 taking priority’.

The first theme, ‘Escaping the external gaze’ emphasises the anxiety that many individuals with a facial visible difference felt around everyday interactions and the difficult dilemma that lockdown presented; with many participants feeling both relief at reduced interactions with others, as well as anticipating future anxiety around returning to normal activities. However, the second theme ‘Existing feelings manifesting in new challenges’ suggests that lockdown was not without challenge for many individuals and new behaviours associated with the COVID-19 pandemic may have been particularly difficult for individuals with a facial visible difference. Some participants suggested that increased use of video conferencing and the ‘2-metre rule’ when in public, as well as the anticipation of compulsory face coverings, were new challenges to navigate, and their feelings around these behaviours were often influenced by their existing adjustment and feelings regarding their visible difference. Finally, ‘COVID-19 taking priority’ reflects a sentiment expressed by many participants, that COVID-19 had shifted their personal focus off their visible difference, as they focussed on other issues. However, many were concerned about the long-term impact on care and support provision for individuals with a visible difference, and thus taking attention away from visible difference was not always thought to be a good thing.

Our findings highlight important considerations for the provision of care and support for people with visible differences during the COVID-19 pandemic, particularly as various restrictions continued in the U.K. for a significant amount of time beyond the first lockdown in March, 2020. Potential care and support that may be helpful for this group include support around increased social interactions after periods of social distancing and managing associated anxiety, and encouraging individuals to seek support or treatment relating to their visible difference and reassuring them regarding the availability of this, as appropriate. Social skills training has been a component of many interventions to support people whose lives have been negatively affected by visible difference (Harcourt et al., 2018; Jenkinson et al., 2015; Norman & Moss, 2015;), and it may be that these interventions are a beneficial tool when supporting individuals with a visible difference who are experiencing anxiety around an increase in social interactions.

The current situation may also be an opportunity for online interventions and screen-based support for individuals with a visible difference, and support organisations may wish to consider the use of evidence-based online interventions to support this population (e.g., ‘Face It’, see Bessell et al., 2012). Similarly, increased use of video-conferencing by the general population may present an opportunity to run support groups online and connect individuals from a wide geographical area, and many support organisations have worked hard to quickly provide this online support to their populations, as well as guidance on adapting to these new methods of interaction. However, support organisations and healthcare professionals should also be aware that the use of teleconferencing may be challenging for some individuals. For example, some participants in this study described these technologies as bringing an increased focus to their appearance, for both themselves and others. This may impact on individuals’ engagement with services and could further impact on their anxiety. Further research would benefit from investigating the use of online interventions, such as those discussed above, during this time and whether individuals with a visible difference feel that these are an acceptable means of support during this time. Regardless of the mode of support, this research has highlighted the importance of support provision for this population and awareness of the impact of the pandemic on this population. This echoes the findings of other studies focused on specific populations within the visible difference community and, as Bruce et al. (2021) wrote, the “importance of remaining vigilant regarding the care of this vulnerable population during this challenging time” (pg. 3).

Importantly, we note that not all participants in this study were struggling with issues related to having a facial difference, and those who described feeling well adjusted to their visible difference prior to lockdown suggested that they had experienced fewer challenges adapting to new behaviours. This aligns with the existing visible difference literature that has demonstrated the complexity and variety in adjustment to living with an unusual or altered appearance (Egan et al., 2011). This variation in adjustment can be seen reflected in the participants who took part in this study and could be said to explain the variation in responses and attitudes observed in the data.

An important area of further research is the experiences of young people with visible differences in the COVID-19 pandemic. This study focused on the experiences of adults in the U.K., yet young people have also experienced significant disruption to their everyday lives, with home schooling reducing contact with usual school friends and support networks and many social interactions moving online. A recent systematic review and meta-analysis suggested that young people with a visible difference may experience more symptoms of anxiety than their peers (van Dalen et al., 2020), and thus the impact of these changes is an important topic for future study and may highlight a role for alternative and additional support.

In addition, a topic that remains salient and warrants further research in both adult and adolescent populations is the experience of wearing a mask in public, and this is another topic that support organisations have worked quickly to provide support around. Many of the sample in this study discussed wearing a mask in terms of their anticipated feelings and had not experienced this at the time of interview, as masks had not yet been made a compulsory element of restrictions. It is possible that their concerns might be allayed as face coverings have become more normalised or perhaps anxiety may have been heightened due to mandatory introduction of masks. Bogart (2021) described covering her facial difference with a mask as being essential but a betrayal of her identity, and this reflects the complex feelings that many participants expressed with regards to mask wearing and other social distancing measures. Bogart (2021) also suggested that mask wearing offers an opportunity to educate people without facial differences and uses facial paralysis as an example. They suggested that wearing a mask may increase individuals’ awareness about the experiences and challenges facing people who have facial paralysis, and therefore potentially destigmatize people with facial paralysis and other disabilities by learning from their adaptations. However, it is important to also recognise the differences between this experience and living with facial paralysis, as well as to be aware of the potential unintended consequences of such disability simulations - “Wearing masks may lead people to feel pity for people with facial paralysis, unless they consider the creative ways people with limited facial expression adapt” (Bogart, 2021; page 842). Further research with a U.K. population of adults and young people with a visible difference regarding their experiences and feelings about masks would be a valuable addition to this literature and provide further insight about how to best support this group as restrictions continue.

## Limitations

Firstly, the sample included in this study were self-selecting and could be suggested to represent a group of individuals who are motivated to take part in research by particularly strong feelings on the topic of study, as is a limitation in many studies with similar volunteer samples. However, the authors feel that the presence of a financial incentive to take part in this study encouraged participation by individuals with a range of views and experiences regarding the topic, and this is reflected in the findings of the study; with some individuals reporting very few challenges with regards to their visible difference. The majority of the participants were, however, female and White British, and all participants were under the age of 65. Thus, this research could be said to be more representative of the experience of White British working-age women with a visible difference. This research did not explicitly seek to explore the role of gender, ethnicity or age in these participants’ experiences, and therefore further research may benefit from exploring this topic with a more diverse sample. Equally, research in the field of visible difference has been acknowledged to often under-represent the experiences of men and therefore further research specifically exploring the experiences of men would also be beneficial (Zucchelli et al., 2022).

Secondly, the authors acknowledge that the results of this study reflect the experiences of the participants at a specific point in time during the COVID-19 pandemic, and significant developments have occurred since the data were collected (May-June, 2020). Although the themes identified offer valuable insight into the experiences of individuals at this time and remain relevant beyond the specific circumstances they refer to, the temporal context in which participants were taking part in this study should be considered and further research would benefit from exploring how individuals’ experiences have changed and evolved through the course of the pandemic.

## Conclusion

The results of this study suggest that the first U.K. lockdown from the COVID-19 pandemic created significant challenges for some individuals with a facial visible difference, and ongoing restrictions and social distancing measures might be particularly challenging for those who experience anxiety around social encounters. This study was conducted in the U.K. at a time when lockdown restrictions were gradually being eased, but before a vaccine for COVID-19 was available. The pandemic is a dynamic and uncertain situation and the participants in this study will have since been subjected to further lockdowns and subsequent easing of restrictions. Support and intervention to prepare people with visible differences for return to greater public interaction may be a valuable tool as restrictions change over time, as well as further research to understand the potential implications for young people with a visible difference and of ongoing use of masks in public. We hope that this research will provide valuable insight for healthcare providers and support organisations working with this group, and potentially inform future support and intervention development in the U.K.

## Data Availability

Due to the nature of this research, participants of this study did not agree for their data to be shared publicly, so supporting data is not available.
